# Next-generation sequencing technology for detecting pulmonary fungal infection in bronchoalveolar lavage fluid of a patient with dermatomyositis: a case report and literature review

**DOI:** 10.1186/s12879-020-05341-8

**Published:** 2020-08-17

**Authors:** Kaiyu Zhang, Chen Yu, Yuxiang Li, Yang Wang

**Affiliations:** 1 Department of Infectious Diseases, the First Hospital, Jilin University, Changchun, 130021 Jilin China; 2 Department of Pediatrics, University of Oklahoma Health Sciences Center, Oklahoma City, OK 73104 USA

**Keywords:** Next-generation sequencing, Bronchoalveolar lavage fluid, Pneumonia

## Abstract

**Background:**

Invasive fungal pneumonia is a severe infectious disease with high mortality in immunocompromised patients. However, the clinical diagnosis of the pathogen(s) remains difficult since microbiological evidence is difficult to acquire.

**Case presentation:**

Here, we report a case of pulmonary fungal infection detected by next-generation sequencing (NGS) of bronchoalveolar lavage fluid (BALF) in a 61-year-old male with corticosteroid-treated dermatomyositis. Cytomegalovirus and influenza A virus infections were confirmed by nucleic acid detection and treated with antiviral medicine. The patient had been diagnosed with severe pneumonia and treated with empiric broad-spectrum antibacterial and antifungal drugs before bronchoscopy was performed. The patient responded poorly to those empiric treatments. Three fungi were found by NGS in the BALF, namely, *Pneumocystis jirovecii*, *Aspergillus fumigatus* and *Rhizopus oryzae*. After adjusting the patient’s treatment plan according to the NGS results, he improved significantly.

**Conclusions:**

This case highlights the combined application of NGS and traditional tests in the clinical diagnosis of pulmonary invasive fungal disease. NGS is proposed as an important adjunctive diagnostic approach for identifying uncommon pathogens.

## Background

Dermatomyositis (DM) is a rare chronic autoimmune disease primarily affecting skeletal muscle and the skin, with characteristic cutaneous findings and varying amounts of systemic involvement [1]. It is still a poorly understood multisystem disease. DM treatment usually involves corticosteroids and immunosuppressants, but the course of these treatments is very long [2]. Following treatment with corticosteroids, the incidence of invasive infection increases, especially pulmonary invasive infection [3]. The causative agents of these invasive infections are difficult to identify because many of the pathogens are ‘unculturable’ or ‘difficult to culture’. Multiple methods of detection, such as specific antigen or antibody detection, PCR amplification, inflammatory indexes and imaging examinations, have been utilized in clinical work, but we occasionally still cannot draw a correct conclusion according to these tests. However, next-generation sequencing (NGS), a highly sensitive method for analyzing the microbiome, could provide additional valuable information for diagnosis and treatment.

Here, we report one patient with DM who developed severe pneumonia and was infected with multiple pathogens, including viruses and three different fungi, and pulmonary fungi were revealed by NGS of bronchoalveolar lavage fluid (BALF).

## Case presentation

A 61-year-old male presented to the infectious disease department of the First Hospital, Jilin University, with fever for 7 days. This patient had been diagnosed with DM 2 months prior. He was prescribed oral methylprednisolone therapy (morning dose 40 mg, night dose 24 mg, daily) without antibiotic prophylaxis after his diagnosis. He came to the hospital because he had a fever with a temperature up to 38.8 °C for 7 days. He also complained of intermittent productive cough with pale sputum associated with chest heaviness, pectoralgia and dyspnea at rest.

**Fig. 1:**
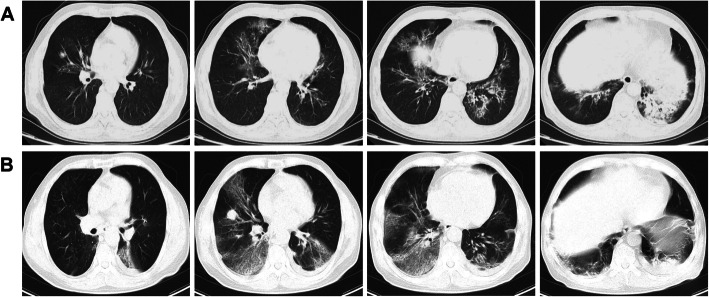
CT scan of the lung. **a** On the 1st day, a CT scan showed bronchitis and inflammation in the right lung lobes, the left ligule lobe and the left lower lobe. **b**: On the 9th day, a CT scan showed that some of the inflammation was alleviated, but inflammation in most lung lobes was worse

The laboratory results were as follows: white blood cell count 11.98 × 10^9^/L, with a neutrophil ratio of 93%; hemoglobin 133 g/L; and platelet count 139 × 10^9^/L. Inflammatory marker levels were increased significantly: C-reactive protein (CRP) 174.1 mg/L; erythrocyte sedimentation rate (ESR) 72 mm/h; and procalcitonin 0.05 ng/mL. Arterial blood gas analysis (with O_2_ 5 L/min via nasal catheter) showed pH 7.51; pCO_2_ 37 mmHg; pO_2_ 60 mmHg; HCO_3_^−^ 29.5 mmol/L; BE 6.1 mmol/L; lactate 1.9 mmol/L; and O_2_ saturation 93%. Blood biochemical index results showed that creatine kinase content increased to 746 U/L, creatine kinase isoenzyme content increased to 86.2 U/L, lactate dehydrogenase content increased to 682 U/L, and ɑ-hydroxybutyrate dehydrogenase content increased to 528 U/L. Nucleic acid detection of common respiratory pathogens (including *Mycoplasma pneumoniae*, *Chlamydia pneumoniae*, adenovirus, respiratory syncytial virus, parainfluenza virus, and influenza A and B) was performed, and nucleic acid detection of influenza A virus was positive. Nucleic acid detection of cytomegalovirus showed 4.5 × 10^4^ copies/mL. A computed tomography (CT) scan showed bronchitis and inflammation in the right lung lobes, the left ligule lobe and the left lower lobe (Fig. 1a). All of the blood culture, urine culture and sputum culture results were negative. Both the 1,3-beta-D-glucan test (G test) result (15 pg/ml) and galactomannan test (GM test) result (0.33 μg/L) were negative. We also sent the sputum sample to perform Gomori-Grocott methenamine silver nitrate staining (GMS) for *Pneumocystis jirovecii* detection and obtained a negative result. The diagnosis of this patient included pneumonia, influenza A virus infection and cytomegalovirus infection.

**Fig. 2:**
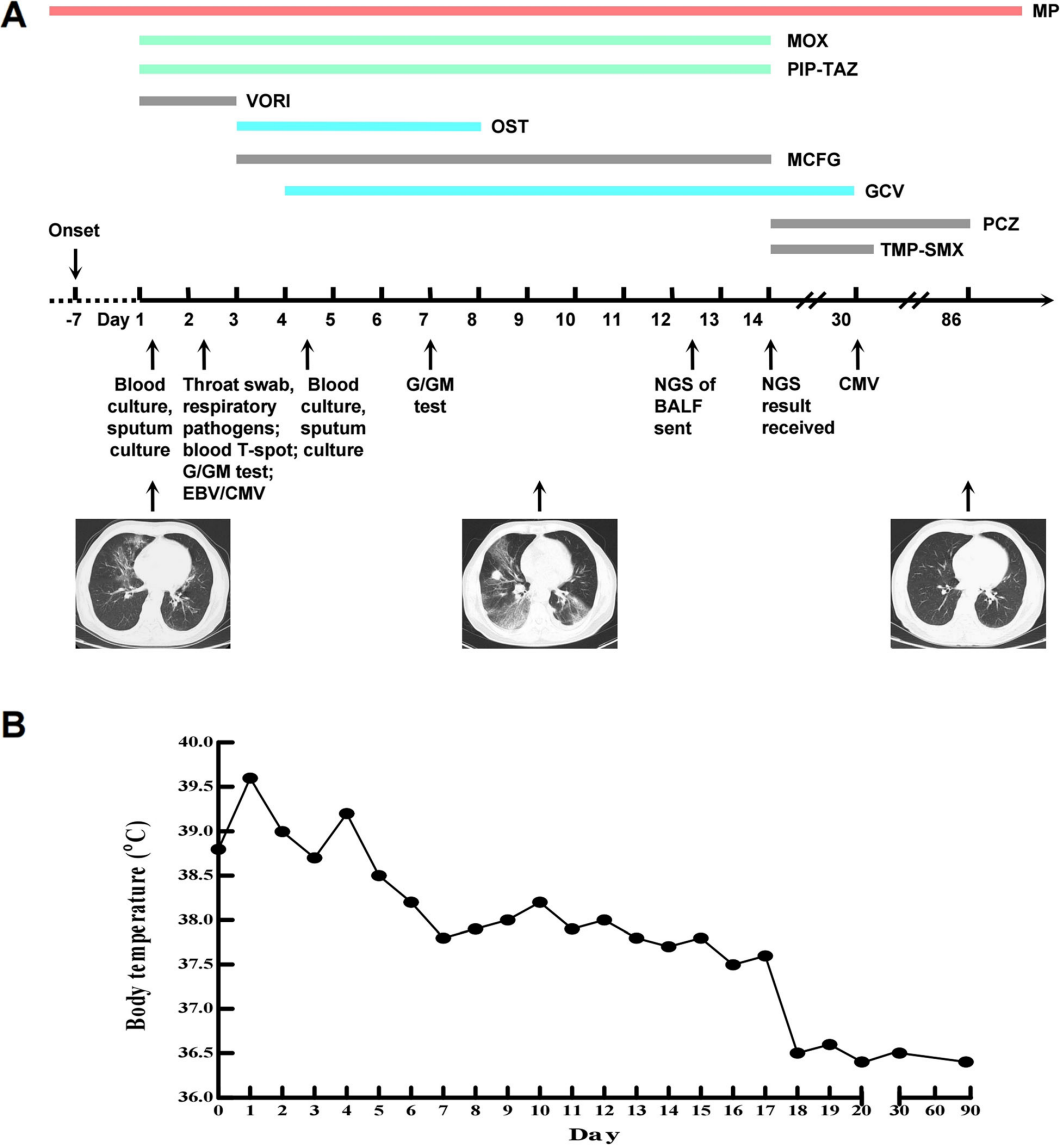
Timeline of the patient’s clinical manifestations and treatment. **a** Timeline of the patient’s tests and treatment. MP, methylprednisolone; MOX, moxifloxacin; PIP-TAZ, piperacillin/tazobactam; OST, oseltamivir; VORI, voriconazole; MCFG, micafungin; GCV, ganciclovir; PCZ, posaconazole; TMP-SMX, trimethoprim/sulfamethoxazole; G test, 1,3-β-D-glucan test; GM test, galactomannan test. **b** Timeline of the patient’s body temperature

**Fig. 3:**
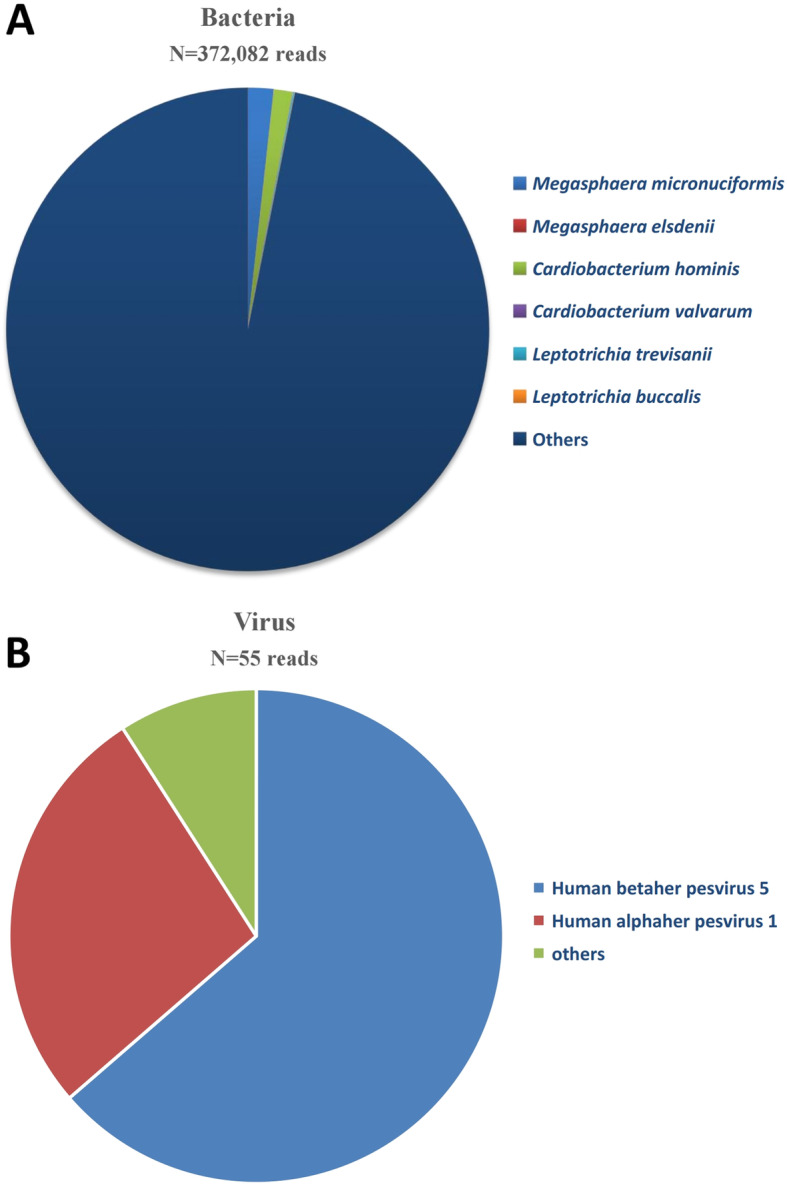
Other microorganisms found by NGS. **a** Bacteria found by NGS. *Megasphaera micronuciformis*: 6358 reads; *Megasphaera elsdenii*: 4 reads; *Cardiobacterium hominis*: 4660 reads; *Cardiobacterium valvarum*: 71 reads; *Leptotrichia trevisanii*: 339 reads; *Leptotrichia buccalis*: 238 reads; others: 360412 reads. **b** Viruses found by NGS. Human betaherpesvirus 5: 35 reads; Human alphaherpesvirus 1: 15 reads; others: 5 reads

Considering the treatment with methylprednisolone and the results of the tests, moxifloxacin (400 mg, QD, iv. D), piperacillin/tazobactam (4.5, Q8H, iv. D) and voriconazole (200 mg, Q12H, iv. D) were used after he was hospitalized (Fig. 2a). Oseltamivir (75 mg, Q12H, orally, for 5 days) was given after the positive result of the nucleic acid detection of influenza A virus was received. After 2 days of treatment with voriconazole, the patient developed visual hallucinations and confusion, and the antifungal treatment was changed to micafungin (50 mg, Q12H, iv. D) (Fig. 2a). Ganciclovir (0.25, Q12H, iv. D) was given to him after receiving the positive nucleic acid detection of cytomegalovirus. On the 6th day after admission to the hospital, his body temperature decreased to below 38.5 °C but did not decrease to normal levels (Fig. 2b). Meanwhile, his cough and dyspnea were obviously relieved. His arterial blood gas analysis (without an O_2_ nasal catheter) showed pO_2_ 80 mmHg and O_2_ saturation 95%. On the 9th day after admission to the hospital, the patient underwent a second CT scan test, and the results showed that some of the inflammation was alleviated, but the inflammation in most lung lobes was worse (Fig. 1b). The G test and GM test results remained negative. Blood biochemical index results showed that the creatine kinase content decreased to 441 U/L, creatine kinase isoenzyme content decreased to 47.5 U/L, lactate dehydrogenase content increased to 505 U/L, and ɑ-hydroxybutyrate dehydrogenase content increased to 395 U/L. To verify pathogen-induced pneumonia, the patient underwent bronchoscopy examination with collection of BALF specimens, and these samples were sent to BGI Diagnosis Co. (Shenzhen, China) for an NGS test on the 12th day after admission to the hospital. We also sent the BALF samples to perform GMS for *Pneumocystis jirovecii* detection, and this time, we obtained a positive result. In addition, another portion of the BALF was sent to the laboratory for traditional culture and T-SPOT, which were reported as negative. Two days later, NGS results showed that three fungi were in the BALF, namely, *Pneumocystis jirovecii*, *Aspergillus fumigatus* and *Rhizopus oryzae* (Table 1). The other microorganisms detected by NGS are listed in Fig. 3, but they were not regarded as responsible for the invasive lung infection. The antifungal therapy was changed to posaconazole (75 mg, Q12H, orally) and trimethoprim/sulfamethoxazole (TMP/SMX, TMP 0.24/SMX 1.2, Q6H, orally, for 3 weeks). The patient improved significantly, and his body temperature decreased to normal levels 4 days after adjusting the treatment plan (Fig. 2b). Then, he was treated with ganciclovir, posaconazole and TMP/SMX at home (Fig. 2a).

**Table 1:** NGS report of the microorganism in BALF

Genus		Species	
Name	Sequence number^a^	Name	Sequence number^a^
*Pneumocystis*	66	*Pneumocystis jirovecii*	66
*Aspergillus*	45	*Aspergillus fumigatus*	11
*Rhizopus*	22	*Rhizopus oryzae*	11

^a^The sequence number of the strict comparison of the microorganism detected at the level of genus/species

The patient’s body temperature remained normal, and no significant symptoms were observed (Fig. 2b). On the 30th day, he underwent nucleic acid detection for cytomegalovirus. The result was negative, and ganciclovir was discontinued. On the 35th day, TMP/SMX was discontinued because its course of treatment was long enough. The latest lung CT scan was taken on the 86th day, and the results showed that the inflammation of the lung lobes had dissipated well. Posaconazole was eventually discontinued (Fig. 2a). Blood biochemical index results were slightly higher than the normal levels but were much better than those before treatment.

## Discussion and conclusions

Corticosteroids are frequently used to treat rheumatic diseases. Their use comes with a number of well-established risks, including glaucoma, avascular necrosis, osteoporosis and diabetes. Corticosteroid treatment could inhibit the host immune system, and immunocompromised patients are easily infected with various opportunistic pathogens [4]. Many studies have suggested that high-dose (> 20 mg/day prednisone) corticosteroids might lead to an increased risk of serious infections, and this increased risk is dose dependent [4]. This patient with DM had been treated with a high-dose corticosteroid for 2 months (methylprednisolone, morning dose 40 mg, night dose 24 mg, daily, orally) before acquiring pneumonia.

It is known that several viruses with different replication mechanisms contribute to oncogenesis in immunosuppressed subjects, both directly and indirectly. Among these viruses, the main ones are as follows: Epstein-Barr virus (EBV), human papillomavirus (HPV), Kaposi sarcoma herpesvirus (KSHV), human T-cell leukemia virus type 1 (HTLV-1), and Merkel cell polyoma virus (MCV) [5]. Worldwide, human cytomegalovirus (CMV) infection is very common, with seroprevalence rates ranging from 40 to nearly 100%. Primary infection is usually subclinical in healthy adults due to a complex antiviral immune response. However, patients receiving immunosuppressive medication are a high-risk group [6]. CMV is the most common infection among immunocompromised patients and has a major impact on morbidity, mortality and graft survival [7]. It has been reported that CMV and other respiratory viral infections are common between 1 and 3 months after immune suppression [8]. In the present study, nucleic acid detection was cytomegalovirus positive, and approximately 4 weeks of ganciclovir therapy was given until the test became negative. Nucleic acid detection of influenza A virus was also positive. The population is generally susceptible to influenza A virus, and this patient did not receive the influenza A virus vaccine. He took oseltamivir for 5 days, with decreasing body temperature.

Infection secondary to corticosteroid therapy could be from bacteria, such as *Mycobacterium tuberculosis*, or fungi [4]. In the present study, the patient’s inflammatory markers were increased significantly; however, the lung CT characteristics did not show specific changes to help us verify the pathogens. The results of blood culture and sputum culture were negative. We used broad-spectrum antibacterials (moxifloxacin and piperacillin/tazobactam) that could kill or inhibit most gram-positive and gram-negative bacteria and atypical respiratory pathogens [9]. In our case, all blood culture, sputum culture, G test and GM test results for this patient were negative. However, invasive fungal infections are generally encountered in immunocompromised patients treated with steroids. *Aspergillus* is a common agent among invasive fungal pathogens, with high mortality, and *Aspergillus* infections most commonly involve the lungs [10]. *Aspergillus fumigatus* is most ubiquitous in the environment and is the major cause of the disease [11]. Considering that voriconazole is the preferred antifungal agent for the primary therapy of invasive *Aspergillus* and is considered the first-line treatment for invasive aspergillosis [12], we chose this medicine even though there was no direct evidence of fungal infection. After the side effects of voriconazole were evident, micafungin was used instead, which is a fungistatic echinocandin and represents an alternative therapy [13]. *Pneumocystis jirovecii* is an opportunistic fungus that can cause interstitial pneumonia in an immunocompromised host. Most reported *Pneumocystis jirovecii* pneumonia (PCP) cases are among HIV-positive or transplant recipients and patients who have hematological malignancies requiring T cell-depleting agents. However, in recent years, PCP has also been found in some patients with autoimmune disease on high-dose glucocorticoids [14, 15]. The diagnosis of PCP depends on a GMS test of sputum to detect cysts and trophic forms of *Pneumocystis jirovecii* and/or nucleic acid detection [16]. These two tests are not regular tests and are not available in most hospitals. These tests are required only in HIV-positive patients, and samples need be sent to a commercial company or laboratory. Microscopic detection of *Pneumocystis jirovecii* in respiratory secretions is simple and useful but may underestimate *Pneumocystis jirovecii* infection. Nucleic acid detection of *Pneumocystis jirovecii* has become increasingly important in recent years [17]. Prophylaxis is highly effective and should be given to all patients at moderate to high risk of PCP [18]. Unfortunately, this patient did not receive prophylaxis for PCP. TMP/SMX remains the drug of choice for prophylaxis and treatment of PCP. *Rhizopus oryzae* exists broadly in the environment and can also be an opportunistic fungus. *Rhizopus oryzae* is one of the causes of mucormycosis [19]. Reported cases of *Rhizopus oryzae* infection are rare, but there have been cases in patients receiving corticosteroid therapy. Among those cases of *Rhizopus oryzae* infection, only one case was related to pulmonary infection [20]. The diagnosis of *Rhizopus oryzae* infection usually depends on fungal culture and lesional biopsy. Posaconazole is a second-generation triazole agent with potent and broad antifungal activity. Posaconazole could be used to treat invasive *Aspergillus* and *Rhizopus* infection. We modified the therapeutic strategies as soon as we obtained the results of NGS and achieved good outcomes.

NGS can be used to sequence the entire DNA/RNA of a sample. The NGS platform we utilized (the BGISEQ-50 sequencing platform) can simultaneously detect a broad range of bacterial, viral, fungal or parasitic DNA/RNA sequences with high accuracy and within less than 30 h [21]. DNA and RNA were extracted from BALF samples. Complementary DNA (cDNA) was generated from an RNA template by reverse transcription. Then, DNA libraries were constructed through DNA fragmentation, end repair, adapter ligation and PCR amplification. Quality qualified libraries were sequenced by using the BGISEQ-50 platform. High-quality sequencing data were generated by removing low-quality and short (length < 35 bp) reads, followed by computational subtraction of human host sequences mapped to the human reference genome (hg19) using Burrows-Wheeler Alignment. The remaining data obtained by removal of low-complexity reads were classified by simultaneous alignment to four microbial genome databases consisting of viruses, bacteria, fungi, and parasites [22, 23]. In recent years, the cost of NGS has decreased, and most patients can afford it. Traditional ‘gold standard’ sample culture requires a much longer time with much lower sensitivity. For the diagnosis of invasive fungal infection, the results of some promising tests (G test and/or GM test) are not specific, and their sensitivity is not satisfactory. In the present case, none of the results of these kinds of tests verified fungal infection. We could only give empiric treatment according to the patient’s medical history, clinical manifestations and examination results until the NGS of BALF result was received; then, the antipathogen therapy was changed to a targeted treatment. NGS can provide direct clues and assist in accurately diagnosing complex infections [24]. Regarding the high mortality associated with severe pneumonia, NGS should be considered a regular test [25]. However, not all microorganisms determined by NGS are associated with disease; for example, NGS can identify colonizing bacteria within the host body. Collectively, clinicians should identify the actual causative agent(s) according to NGS results, the characteristics of the detected pathogen(s) and other examinations results.

NGS is currently not the best choice for fungal identification. However, the diagnosis of invasive fungal infection is very difficult, and NGS in BALF has become increasingly important and is regarded as an important adjunctive diagnostic approach for uncommon pathogens. This case report highlights the feasibility of deploying NGS of BALF as a rapid and sensitive diagnostic assay for severe pneumonia among immunocompromised patients, especially those patients treated with corticosteroids. This method may help clinicians make an accurate diagnosis of invasive pathogens and apply the most appropriate therapy.

## Data Availability

The datasets used and/or analysed during the current study are available from the corresponding author on reasonable request.

## Abbreviations

BALF: Bronchoalveolar lavage fluid
cDNA: Complementary DNA
CMV: Cytomegalovirus
CRP: C-reactive protein
CT: Computed tomography
DM: Dermatomyositis
EBV: Epstein-Barr virus
ESR: Erythrocyte sedimentation rate
G test: 1,3-beta-D-glucan test
GCV: Ganciclovir
GM test: Galactomannan test
GMS: Gomori-Grocott methenamine silver nitrate staining
HPV: Human papillomavirus
HTLV-1: Human T-cell leukemia virus type 1
KSHV: Kaposi sarcoma herpesvirus
MCFG: Micafungin
MCV: Merkel cell polyoma virus
MOX: Moxifloxacin
MP: Methylprednisolone
NGS: Next-generation sequencing
OST: Oseltamivir
PCP: *Pneumocystis jirovecii* pneumonia
PCZ: Posaconazole
PIP-TAZ: Piperacillin/tazobactam
TMP/SMX: Trimethoprim/sulfamethoxazole
VORI: Voriconazole
